# Human Osteoblast Cell Behaviour on Titanium Discs Treated with Argon Plasma

**DOI:** 10.3390/ma12111735

**Published:** 2019-05-28

**Authors:** Carolina González-Blanco, María Rizo-Gorrita, Irene Luna-Oliva, María-Ángeles Serrera-Figallo, Daniel Torres-Lagares, José-Luis Gutiérrez-Pérez

**Affiliations:** Department of Oral Surgery, College of Dentistry, Seville University, Calle de Avicena s/n 41009 Seville, Spain; karol_blank@hotmail.com (C.G.-B.); marrizgor@alum.us.es (M.R.-G.); irenelunaoliva@gmail.com (I.L.-O.); danieltl@us.es (D.T.-L.); jlgp@us.es (J.-L.G.-P.)

**Keywords:** dental implants, osteoblasts/drug effects, argon/therapeutic use, titanium, surface decontamination

## Abstract

(1) Background. Titanium is characterized by its biocompatibility and resistance to stress and fatigue. Treatment with argon plasma may favour growth of human osteoblasts with respect to cell adhesion and proliferation. The aim of this study was to analyse the behaviour of human osteoblasts (MG-63) on Grade IV and V titanium possessing a sand-blasted, acid-etched (SLA) surface. SLA is a widely used surface treatment to create micro- and macroretentions to enhance osteoconductive properties on the surface. (2) Methods. One group of each grade of titanium was decontaminated with argon plasma and compared. On each disc, 20 × 10^4^ cells were cultivated for morphological analysis, study of cell viability (regarding a negative control [100% viability]) and mitochondrial energy balance. (3) Results. At 24 h titanium treated with SLA showed a higher percentage of cell viability (47.3 ± 8.1%) compared to titanium IV treated with argon plasma, which presented a percentage of 79.1 ± 1.1%. Grade V titanium treated with argon plasma presented a higher viability percentage 91.3 ± 3.0% whereas nontreated Grade V titanium presented 53.3 ± 4.0%. Cells cultivated on the surfaces with an argon-plasma treatment were enlarged in comparison to non-treated discs. The cells with smaller circularity with a greater spread and spindle shape were the ones cultivated on the Grade V titanium surface. Cells seeded on treated titanium IV and titanium V, treated or not, showed higher mitochondrial activity over nontreated titanium IV. (4) Conclusions. Cells cultivated on those Grade V titanium discs that were decontaminated with argon plasma presented higher levels of cell adhesion and proliferation, lower mitochondrial damage and a higher mean cell area compared to those not decontaminated with argon plasma.

## 1. Introduction

Oral rehabilitation with dental implants is a common procedure nowadays, and titanium is the main element of which they consist. There are different degrees of titanium in which there is an increase in the elements Fe, O and N as the degree increases. Grade IV titanium is the most commonly used in manufacturing of implants, but also different titanium alloys with other elements (aluminium and vanadium) have been made to achieve Grade V titanium (Ti_6_Al_4_V) with which better mechanical properties are obtained [1].

Several treatments can be applied to the titanium surface to improve its osteoconductive properties by adding or subtracting material and cleaning or removing the native surface layer. Some examples of former techniques are plasma spraying, sandblasting or acid etching [2,3,4,5]. The combination of these two latter techniques leads to sand-blasted, acid-etched (SLA) surfaces, which provide superficial topographic changes at the macro and microscopic levels with an average pore size of 1–3 μm [6]. This technique is widely used due to the improvement in the union of the implant surface with osteoblastic cells, a key factor in the osseointegration of titanium [7].

Once the surface of the implant has been milled and surface treated, an additional step that can be carried out is the decontamination of the surface of the implant before blistering and sterilization with gamma rays [8].

Surface contamination is inevitable and attributed to organic elements such as carbon and impurities from the manufacturing process of the implant that are deposited on its surface. Different kinds of cold plasma treatments, such oxygen, argon or helium, can be applied [9]. In our study, an argon chamber was used. It works by activating the electronic coating of materials using vacuum argon-based plasma treatment. The main effect of the mentioned activation is the removal of organic contamination and contamination from manufacturing. At the same time, this process can also modify surface chemistry and, in turn, the biological characteristics of the implant surfaces and their interaction with the environment [8]. This procedure may be carried out since it has been observed that carbon remains may limit the cell-implant interaction, which results in a lower implant bone union (BIC) [7,10].

Several authors have reported the advantages of this technique when it is applied for a few minutes before implant material utilization. This technique also yielded improved cell adhesion and protein adsorption in in vitro studies [11,12,13,14,15,16].

The aim of this study was to analyse and compare growth, size measurements, viability and the mitochondrial energy balance of MG-63 cells seeded on titanium IV or V discs that were treated (or not treated) with non-thermal argon plasma. The null hypothesis was that there is no difference at the level of cell morphology, spreading, viability or mitochondrial activity between the surfaces.

## 2. Materials and Methods

### 2.1. Sample Preparation

This in vitro study was carried out at the Centre for Scientific Instrumentation (CIC) of the University of Granada to study osteoblast cell growth on different commercially modified Grade IV and V titanium discs (8 mm in width, 2 mm in height). Four different types of discs were used: Grade IV titanium discs with SLA treatment and no argon-plasma treatment (GR4NT); Grade IV titanium discs treated with SLA and argon plasma (GR4TR); Grade V titanium discs treated with SLA, but not treated with argon plasma (GR5NT) and Grade V discs with SLA and argon plasma (GR5TR). Five discs were used for each group.

Treatment with SLA (sand-blasted, large-grit, acid-etched) was based on coarse grain (250 to 500 mm) silicon particles blasted following an acid etching treatment using a mixture of hydrochloric and sulphuric acids on the titanium surfaces, obtaining a surface roughness (R_a_) of 1.5 ± 0.2 µm [16].

A vacuum cold plasma treatment was performed using a V15-G plasma reactor produced by Plasma Finish (PINK GmbH Thermosysteme, Wertheim, Germany), placed into an ISO 7 clean room (Lesatec, Opera Milan, Italy). The machine is equipped with a radio-frequency generator. The gas flow rate is controlled by a caudalimeter MKS Vortex (SIAD Macchine Impianti, Bergamo, Italy), flow meters and Ar (>99.9990% purity). After reaching 10-Pa base pressure, Ar was introduced in the reactor chamber and the treatment was performed using 590 J/mL power-to-flow-rate ratio. The operating pressure was 20 Pa and the treatment time was 15 min.

### 2.2. Cell Culture

The MG-63 cell line was used in this study because it can retain the differentiated phenotype over consecutive subcultures and shows a faster growth than the primary bone-forming lines, which makes it a good in vitro model. MG-63 cells (Sigma-Aldrich, Darmstadt, Germany) were cultivated in T75 culture flasks with Dulbecco’s Modified Eagle Medium (Biowest, Nuaillé, France) enriched with a 1% antibiotic (glutamine-penicillin-streptomycin [Biowest, Nuaillé, France]) and 10% foetal bovine serum (Biowest). After performing two cell subcultures and achieving an 80% confluence, the cells were seeded on the disc surfaces in a 24-microwells plate at a previously calculated concentration of 2 × 10^4^ cells. Three discs were employed for each type of surface and the experiments were triplicated. Microwells without discs were used as negative controls. After 4 h of incubation, the cell culture was controlled by an Olympus CKX41SF2 phase contrast microscope (Olympus, Shinjuku-ku, Tokyo, Japan). Then, the microwells were completely filled with Dulbecco’s Modified Eagle Medium and incubated for a period of 24 h.

### 2.3. Cell Viability Analysis

Cell viability was assessed after 6 h, 24 h and 48 h, on the 4 types of surfaces (GR4NT, GR4TR, GR5NT and GR5TR), in triplicate, to thereby determine cell proliferation. The WST-1 assay (Sigma-Aldrich, Darmstadt, Germany) was used both in the sample and control wells. As a positive control of cell damage, xantine oxidase (XO, Sigma-Aldrich, Darmstadt, Germany) was used. Xantine oxidase is an enzyme that upon contact with cells, releases free radicals and therefore reduces the fluorescence intensity issued by the cells on spectrophotometry. A value of 100% of viability was given to the negative control (cells seeded on wells without discs), and viability values of the samples were obtained as a percentage of this value.

### 2.4. Morphological Analysis and Mitochondrial Energy Balance

For morphological cell analysis and determination of mitochondrial energy balance of the cells that adhered to the discs, osteoblasts were cultured on the discs on the four types of surfaces analysed (GR4NT, GR4TR, GR5NT and GR5TR), in triplicate.

After an incubation period of 24 h, cell staining was performed using Phalloidine-TRITC (Sigma, Darmstadt, Germany), which stains the F-actin of the cell cytoskeleton. DAPI was used (Sigma-Aldrich, Darmstadt, Germany) to stain the nuclei. The images were taken with an Axio Imager Z1 fluorescence microscope (Carl Zeiss, Jena, Germany) with in 40× magnification.

Likewise, cell size measurements were taken with an ImageJ v1.50e (National Institutes of Health, Bethesda, MD, USA). The segmentation tool was employed to detect cell shapes automatically and to measure the cell area and circularity. This latter parameter is dimensionless and based on the formula $\frac{\left(Perimeter\right)2}{4\pi Area}$, perimeter = 2πr, and its value ranges between 0 (elongated shapes) and 1 (perfect circle) [17].

Finally, to evaluate the mitochondrial energy balance of the MG-63 cells, microphotographs were taken after culture staining with JC-1 (Merck KGaA, Darmstadt, Germany). At low concentrations, this dye forms monomers with green fluorescence; at higher concentrations, it forms aggregates with a red emission. This red/green ratio reveals the membrane potential. Images were taken with the Axio Imager Z1 fluorescence microscope (Carl Zeiss, Jena, Germany) and the red/green ratio was measured with the FC500MPL cytometer (Beckman Coulter, Brea, CA, USA).

### 2.5. Statistical Analysis

The comparison of the groups for each of the analysed variables was made using IBM SPSS Statistics 24.0 software (International Business Machines Corp., New York, NY, USA). The Kolmogorov–Smirnov test was performed to verify normal distribution. Homogeneity equality of variance was checked using Levene’s test. One-way analysis of variance (ANOVA) was calculated. The Bonferroni test was applied for comparing the groups. A 5% level of statistical significance was established (p < 0.05).

## 3. Results

### 3.1. Cell Viability

Cell viability percentage was calculated regarding control viability (100%) after 6 h, 24 h and 48 h on the four types of surfaces (Table 1).

The data obtained in this study indicate that cell viability measured after 6 and 24 h was higher on both titanium Groups IV and V treated with argon plasma (statistically significant differences). On the other hand, viability at 48 h was greater on both titanium V groups (treated or not) over titanium Grade IV groups. Nevertheless, no statistically significant differences were observed.

### 3.2. Morphological Analysis of Cells

**Figure 1:**
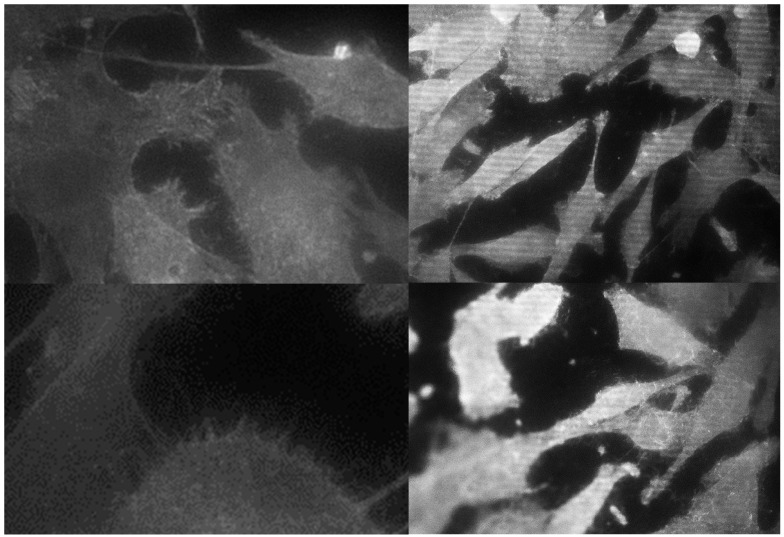
Confocal microscope images of MG-63 on the GR4NT surface (top left), GR4TR (top right), GR5NT (bottom left) and GR5RT (bottom right) at 40× magnification (details, approximately 150×). Philopodia-like cell processes can be observed in the images.

Fluorescence microphotographs were used for the morphological analysis after 24 h of incubation. MG-63 showed philopodia-like cell processes that indicate the cell adhesion to the surface (Figure 1).

Both argon plasma groups showed a greater average cell area in comparison with those without cold plasma treatment.

Statistically significant differences were found between the GR4NT-GR5TR and GR5NT-GR5TR groups. Cells cultivated on the surfaces with an argon-plasma treatment were enlarged in comparison to cells on non-treated discs. Regarding circularity values, the values on the GR4NT surface were larger compared to the other groups. This was interpreted as more round cells and, therefore, less extended or adapted on the surface without treatment with argon plasma. No statistically significant difference was found in the group that did not receive decontamination treatment (Table 2).

Regarding cell area, statistically significant differences were found between groups GR4NT-GR5TR, GR4TR-GR5TR and GR5NT-GR5TR. The cells with smaller circularity values (and therefore with a greater spread and spindle shape) were the ones cultivated on the Grade V titanium surface.

### 3.3. Analysis of Mitochondrial Energy Balance

**Figure 2:**
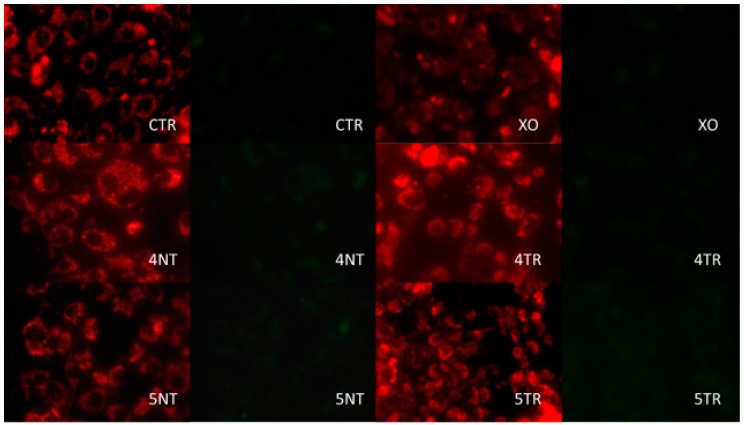
Fluorescence microscopy. View of the accumulation of mitochondrial staining in red and green 24 h after seeding (40× magnification).

**Figure 3:**
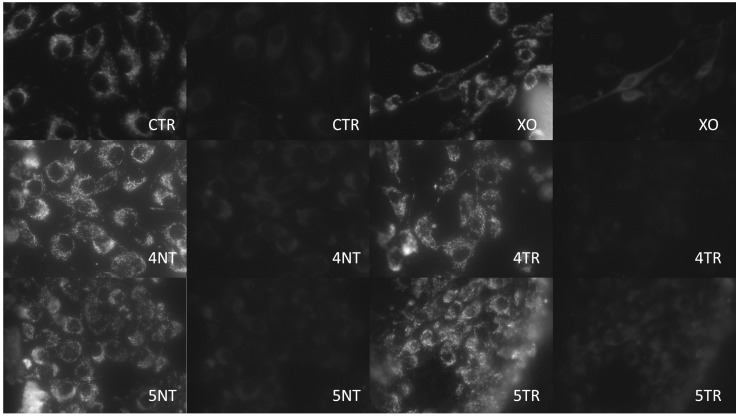
Fluorescence microscopy. Microphotography of the mitochondrial stain in the negative controls (CTR) and positive ones with xanthine oxidase (XO). Mitochondrial damage to MG-63 cells on the 4TR was higher than in the 4NT group; higher mitochondrial damage was observed on the 5NT group compared to the 5TR group (40× magnification).

Mitochondrial energy balance was analysed on the four surfaces using green and red filters (Table 3) and confocal fluorescence microscopy (Figure 2 and Figure 3).

The ratio was calculated using the results obtained with the cytometer. Higher mitochondrial activity was observed in the MG-63 cells in the GR4TR than in the GR4NT group; nevertheless, lower mitochondrial activity was observed in the GR5TR than in the GR5NT group, with the GR5TR group’s energy balance similar to the negative control.

## 4. Discussion

Environmental contamination of the surface of dental implants, prior to their placement, by particles such as carbon, sodium or calcium is an inevitable fact, which involves, according to the literature, a decrease in osteoconductivity or protein adsorption and, consequently, a decrease in the maximum contact of the implant surface with the bone [7]. Furthermore, it is known that this deposition of carbon impurities is incremental over time until saturation [11]. Several techniques have been developed in the last few years, such as decontamination using UV (ultraviolet) rays [18,19] or cold plasma or non-thermal plasma treatments to improve cell response on the surface [15].

The objective of our research was to study whether decontamination with argon plasma led to greater cell viability compared with the non-decontamination of Grade IV and V titanium implant surfaces with SLA treatment. Furthermore, other cell parameters were assessed, such as cell area and circularity, which could indicate the degree of cell adaptation on the surfaces we studied. Finally, the mitochondrial energy balance was analysed as a measure of cell stress and apoptosis.

There are several studies in the literature that have analysed the effect of argon-plasma treatment on titanium surfaces, which has been widely used in recent years, mainly due to its versatility [20].

In this study, it was observed that cell viability after 6, 24 and 48 h was higher on titanium surfaces that were decontaminated with argon plasma. No statistically significant differences were found between the groups after 48 h and this could be due to cell confluence. The viability values found on the titanium IV argon-treated group are similar to the ones found by Rizo-Gorrita et al., who conducted a study on Grade IV titanium implants with SLA surfaces in which treatment with argon plasma was employed and compared with machined surfaces [16]. MG-63 cells were seeded, and cell viability was measured after 24, 48 and 96 h. At 24 h, a statistically significant difference was observed between both titanium type groups; viability was higher in the surfaces treated with argon plasma. No statistically significant differences were found after 48 and 96 h.

In that study, XPS (X-ray photoelectron spectroscopy) analysis (PHI 5400 ESCA Perkin Elmer, Waltham, MA, USA) was done and it showed a lower carbon content after argon-plasma treatment. This study completes the latter from a morphological and cellular activity point of view. From both studies it could be interpreted that argon treatment leads to a lower content of contamination particles on the titanium surface and it improves cell viability, leading to better cell spreading on the surface and higher mitochondrial activity.

Other authors have also compared surface treatment with argon plasma with techniques such as oxygen plasma or UV radiation. Henningsen et al. compared surface decontamination techniques using UV radiation, oxygen or argon plasma with respect to carbon deposition, viability and cell growth after 48 h. It was found that none of the techniques changed the surface of the discs; however, they did improve their wettability, showing a significant difference of the group compared to the control, but no significant difference between argon plasma and oxygen plasma was found. Furthermore, a reduction in the carbon deposition levels was observed with the three treatments. At the same time, an increase in the thickness of the layer of titanium oxide was observed, which was greater after decontamination with argon plasma. Nevertheless, better anchorage and cell growth was observed at 48 h on the discs treated with argon plasma compared to those treated with oxygen plasma, UV radiation and control. Overall, the authors reported that cells on discs treated with argon plasma showed slightly better behaviour than cells on discs treated with oxygen plasma concerning cell attachment and cell viability [12]. Choi et al. obtained similar results [21]. In our study, no differences were observed between groups in terms of viability at 48 h and this could be because of the stabilization of the cell culture reaching confluence.

On the other hand, Smeets et al. obtained higher cell proliferation and viability after 24 and 48 h with cold atmospheric oxygen plasma compared to cold atmospheric argon plasma when osteoblasts were seeded on a zirconium surface. According to the authors, this phenomenon could be explained by the fact that the wavelength used in oxygen plasma (240 nm) is closer to the wavelength needed for expressing the photocatalytic activity of the zirconia surface (213 nm). UV light and argon plasma generated further wavelengths (about 250 nm) [22]. In another study from Henningsen et al., Grade IV titanium and zirconia discs were treated with UV radiation or oxygen or argon plasma. There were no changes in roughness, but non-thermal plasma groups achieved better parameters regarding wettability and XPS surface analysis. Argon plasma may offer the best treatment to obtain the best surface wettability, highest titanium oxide layer and lowest carbon deposition on the surfaces [23]. This could explain the improved morphological cell parameters with higher cell area and higher spreading.

Canullo et al. and Coelho et al. studied the effect of argon-plasma treatment on early biological response using different surface modification techniques on dental implants. Non-thermal plasma of argon (NTP) showed a positive response in terms of osteoblast adhesion and protein adsorption. Within its limitations, the study highlighted the potential benefits of implant surface treatment with argon plasma (12 min) or UV (3 h) [15,24]. In our study, philopodia-like cell processes were observed indicating the cell adhesion to the surface, being smaller than those seeded on non-treated titanium.

De Queiroz et al. analysed the effect of argon-plasma treatment on grade II titanium surfaces regarding the quantification of cell viability by comparing it with untreated surfaces. A viability of 72 h in excess of 80% was obtained on the treated surfaces, as well as better cell adhesion on increasing the hydrophility of the surface with argon-plasma treatment [25]. Our study was undertaken under similar experimental parameters and similar results were obtained at 48 h, with viability reaching 80% in the majority of the groups.

In the study by Tavares et al., which was conducted on titanium discs using polished commercially pure titanium (Grade II, argon-plasma-treated titanium surfaces showed less genotoxic effect) [26].

Concerning quantification of the measurement of cell size, a greater average cell area (µm^2^) was obtained in titanium V with argon plasma than in the rest of the groups; those cells seeded on SLA surfaces treated with argon plasma were larger. From this result, it may be interpreted that this treatment improves the surface by favouring greater cell spreading.

Regarding circularity, lower values were obtained in the titanium V (GR5NT and GR5TR) groups, with a greater spread and spindle shape. Cells seeded on titanium IV were rounder and therefore less spread over the surface, meaning a better cell behaviour on this surface. Similar results were obtained by other authors decontaminating the surface with argon plasma applied using microwaves. It was observed that preosteoblastic cells cultivated on SLA-treated titanium were greater in number and area, showing more spread within cells than those cultivated on untreated surfaces on which cells were seen to be rounder and smaller [27].

Pistilli et al. assessed protein adsorption and cell adhesion on four types of titanium discs in which argon plasma was compared with a control. An increase in levels of protein adsorption and cell adhesion was observed in all groups treated with argon plasma, being those discs with a rougher surface in which higher values were observed [28].

Naujokat et al. conducted an in vivo study on which implant surfaces were prepared with argon plasma for 4 min prior to insertion into pig bone. Neither macroscopic nor microscopic changes were observed after treatment. The primary stability of the implants was not affected. Bone-to-implant contact (BIC) values were investigated. Higher BIC values and bone density were found in those implants that had been prepared in advance. The authors claimed that this technique could be performed routinely by the specialist using a pen-type plasma device before inserting the implant but the effect could be lower than an argon chamber method [29].

In a study performed by Annunziata et al. [8], the results suggested that argon-plasma treatment could be used efficiently for decontaminating previously infected titanium implant surfaces. These encouraging results may be extended to the decontamination of infected implant surfaces, in order to stimulate a favourable response in hard and soft periimplant tissues.

In the study performed by Duske et al., the potential of cold argon plasma complemented by 1% oxygen was investigated for treating rough titanium surfaces contaminated with plaque biofilms. To classify the results, treatment with mechanical cleaning (brushing) was included. Neither a single treatment with CAP (cold atmospheric plasma) nor a single brushing provided a completely decontaminated surface. Consequently, osteoblast cells could not develop normally in the presence of bacteria. A combined treatment of brushing followed by CAP treatment led to a clean surface, thus enabling the cells to grow in a comparable fashion to those cells in the sterile control. Therefore, the synergetic antimicrobial effect of the combined application with brushing and CAP seems to be a promising strategy for decontaminating implants with a rough titanium surface [30].

Once again, there is evidence in a study performed by Teixeira et al. that treatment of dental implants with argon plasma at room temperature can produce substantial improvements in biomechanical fixation at the early stages of implantation, improving osseointegration [31].

In a study by Garcia et al., several cleaning processes were carried out that produced different levels of cell adhesion to the surfaces of the implant abutments. From a descriptive perspective, implant abutments cleaned with argon plasma showed greater cell adhesion than the control group (commercially available abutments without additional treatment). The effect of argon-plasma treatment was also assessed as influential on the distribution of soft tissues. The area occupied by the cells was higher in the plasma group than in the untreated group, with a statistically significant difference. Furthermore, there was no bacterial contamination in 100% of the abutments of the decontaminated group, while in the control group, 40% presented surface contamination. Besides, a biopsy of the gum around the abutments was taken to study the orientation and density of the collagen fibres, those in the plasma group having greater density with a predominance of oblique fibres [32].

In another study, Canullo et al. suggested that the removal of contaminating organic particles from titanium dental implants using argon plasma may enable better maintenance of the bone level compared to steam cleaning for 5 s. The importance of using clean and sterile dental implants in clinical practice became clear [33].

We can say that after comparing titanium surfaces treated or not with argon plasma, significant changes were observed in cellular behaviour and therefore it is necessary to reject the null hypothesis of equal cellular response. Cell viability was increased in cells cultured on treated surfaces at 6 and 24 h, reaching stability in the viability between all groups at 48 h due to the cell confluence. On the other hand, cell size was larger in those cells seeded on treated surfaces and generally with low circularity values with respect to the untreated groups, being interpreted as better cell spreading on the surface. In this study, an assay was performed to determine mitochondrial energy balance using JC-1 staining after 24 h of incubation. This type of analysis has been carried out by other authors on different types of titanium surfaces [34]. The highest mitochondrial activity was observed in the titanium V group without argon treatment. Comparing both argon-plasma-treated titanium groups, cells seeded on titanium IV showed higher activity than titanium V. The results of mitochondrial energy activity should be analysed in conjunction with the results of the other variables that were increased.

The comparison between both types of titanium treated with argon plasma should be cautious. The materials are different. According to our study, GR5TR presented a better overall cellular behaviour with respect to GR4TR. As both materials are suitable to use within the field of oral implantology, there are studies that show a best long-term performance on Grade IV than Grade V titanium, respectively [35]. Nevertheless, other authors did not find differences between these types of titanium [36,37]; for this reason, it would be of interest to verify these results in further studies. Some limitations of this study could be the number of analysed samples and the cell line employed. MG-63 is a tumoural cell line and could express a progressive heterogeneous phenotype due to continued subcultivation. In addition, it could show a nonphysiologic proliferation, not reflecting the whole phenotype characteristics of primary osteoblasts [38]. It is for this reason that the generalisability of the data provided in this study should be taken with caution, being the main factor to emphasize the improvement of the general cellular behaviour after the treatment with argon plasma in the titanium surfaces.

## 5. Conclusions

Considering the results that we obtained in our study, argon-plasma- treated titanium V discs showed higher levels of cell adhesion and proliferation of MG-63 cells, lower mitochondrial damage and a larger cell area (µm^2^) compared to non-treated Grade V titanium and Grade IV titanium (treated or not). Grade IV titanium is the most commonly used type of titanium, and it showed an improvement in cellular behaviour and greater cell size on surfaces treated with argon plasma. One of the limitations of this study was the number of analysed samples and cell line used, but the data of this study indicate that the use of argon plasma as an intervention for decontaminating the surfaces of titanium implants may lead to an improvement in the growth, cell size, spreading and mitochondrial activity of the MG-63 cells that cover them. Future in vitro and clinical studies should corroborate these experimental results.

## Figures and Tables

**Table 1:** Cell viability percentage was calculated regarding control viability (100%) on the various surfaces analysed according to the time slots established.

Sample	Titanium IV (GR4NT)	Titanium IV (GR4TR)	Titanium V (GR5NT)	Titanium V (GR5TR)
6 h	38.7 ± 2.5% ^ab^	79.8 ± 15.2% ^a^	53.3 ± 4.0%	87.6 ± 6.3% ^b^
24 h	47.3 ± 8.1% ^ab^	79.1 ± 1.1% ^a^	77.0 ± 11.0% ^c^	91.3 ± 3.0% ^bc^
48 h	86.0 ± 19.3%	77.2 ± 2.5%	93.0 ± 5.2%	105.4 ± 3.5%

^1^ Note: In the same row, the pairs of identical letters (a-a, b-b, …) identify values with a statistically significant difference (p < 0.05).

**Table 2:** Cell area and circularity values obtained on the four surfaces analysed.

Cell Parameter	Titanium IV (GR4NT)	Titanium IV (GR4TR)	Titanium V (GR5NT)	Titanium V (GR5TR)
AREA (µm^2^)	430 ± 208 ^a^	1212 ± 412 ^b^	525 ± 184 ^c^	1474 ± 425 ^abc^
CIRCULARITY	0.42 ± 0.02 ^ab^	0.38 ± 0.03 ^c^	0.30 ± 0.03 ^acd^	0.34 ± 0.01 ^b^

^1^ Note: In the same row, the pairs of identical letters (a-a, b-b, …) identify values with a statistically significant difference (p < 0.05).

**Table 3:** Results of the determination of mitochondrial energy balance with JC-1 staining after 24 h of incubation.

	Titanium IV (GR4NT)	Titanium IV (GR4TR)	Titanium V (GR5NT)	Titanium V (GR5TR)	Control (CTR)	Xanthine Oxidase (XO)
**JC 1**	4.56 ± 0.58 ^abcd^	11.88 ± 0.60 ^a^	13.40 ± 1.04 ^be^	10.93 ± 2.66 ^cef^	13.2 ± 1.7 ^df^	3.7 ± 0.5 *

^1^ Note: In the same row, the pairs of identical letters (a-a, b-b, …) identify values with a statistically significant difference (p < 0.05). * XO group showed statistically significant differences from all the groups.
